# Safety application of muscle relaxants and the traditional low-frequency ventilation during the flexible or rigid bronchoscopy in patients with central airway obstruction: a retrospective observational study

**DOI:** 10.1186/s12871-021-01321-w

**Published:** 2021-04-06

**Authors:** Jing-Jin Li, Nan Li, Wei-Jia Ma, Ming-Xue Bao, Zi-Yang Chen, Zheng-Nian Ding

**Affiliations:** Department of Anesthesiology and Perioperative Medicine, The First Affiliated Hospital with Nanjing Medical University, Nanjing, 210029 Jiangsu China

**Keywords:** Anesthesia management, Bronchoscopy, Central airway obstruction, Muscle relaxants, Traditional low-frequency ventilation

## Abstract

**Background:**

Bronchoscopy treatments of central airway obstruction (CAO) under general anesthesia are high-risky procedures, and posing a giant challenge to the anesthesiologists. We summarized and analyzed our clinical experience in patients with CAO undergoing flexible or rigid bronchoscopy, to estimate the safety of skeletal muscle relaxants application and the traditional Low-frequency ventilation.

**Methods:**

Clinical data of 375 patients with CAO who underwent urgent endoscopic treatments in general anesthesia from January 2016 to October 2019 were retrospectively reviewed. The use ratio of skeletal muscle relaxants, dose of skeletal muscle relaxants used, the incidence of perioperative adverse events, adequacy of ventilation and gas exchange, post-operative recovery between rigid bronchoscopy and flexible bronchoscopy therapy, and risk factors for postoperative ICU admission were evaluated.

**Results:**

Of the 375 patients with CAO, 204 patients were treated with flexible bronchoscopy and 171 patients were treated with rigid bronchoscopy. Muscle relaxants were used in 362 of 375 patients (including 313 cisatracurium, 45 rocuronium, 4 atracurium, and 13 unrecorded). The usage rate of muscle relaxants (96.5% in total) was very high in patients with CAO who underwent either flexible bronchoscopy (96.6%) or rigid bronchoscopy (96.5%) therapy. The dosage of skeletal muscle relaxants (Cisatracium) used was higher in rigid bronchoscopy compared with flexible bronchoscopy therapy (10.8 ± 3.8 VS 11.6 ± 3.6 mg, respectively, *p* < 0.05). No patient suffered the failure of ventilation, bronchospasm and intraoperative cough either in flexible or rigid bronchoscopy therapy. Hypoxemia was occurred in 13 patients (8 in flexible, 5 in rigid bronchoscopy) during the procedure, and reintubation after extubation happened in 2 patients with flexible bronchoscopy. Sufficient ventilation was successfully established using the traditional Low-frequency ventilation with no significant carbon dioxide accumulation and hypoxemia occurred both in flexible and rigid bronchoscopy group (*p* > 0.05). Three patients (1 in flexible and 2 in rigid) died, during the post-operative recovery, and the higher grade of American Society of Anesthesiologists (ASA) and obvious dyspnea or orthopnea were the independent risk factors for postoperative ICU admission.

**Conclusion:**

The muscle relaxants and low-frequency traditional ventilation can be safely used both in flexible and rigid bronchoscopy treatments in patients with CAO. These results may provide strong clinical evidence for optimizing the anesthesia management of bronchoscopy for these patients.

## Background

The quality of life of the patients is seriously impaired by severe CAO presented as severe dyspnea, stridor, or even respiratory failure. CAO is a potentially life-threatening condition, which has been treated in many ways [1, 2]. For patients amenable to surgery, resection and reconstruction is the best therapeutic option. However, whenever surgery is not feasible, endoscopic therapies are needed [3, 4].

Nowadays, endoscopic treatment has been widely used as an effective method to treat CAO, palliating dyspnea in some cases of malignant obstruction and even be curative in some cases of benign tumor or inflammation [5–7]. Such procedures are mainly performed using rigid or flexible bronchoscope.

These interventions are high-risky procedures, posing a giant challenge to the anesthesiologists. How to establish adequate gas exchange to maintain the life of patients and allow good surgical access is what should be considered during anesthesia [6, 8]. Furthermore, the choice of ventilation strategy and the use of skeletal muscle relaxants are still significant issues for anesthesiologists to consider.

In this article, we summarized and analyzed our clinical experience in anesthesia management in patients with CAO undergoing flexible or rigid bronchoscopy in general anesthesia from January 2016 to October 2019, including the use of muscle relaxants and the traditional Low-frequency ventilation, to estimate the safety of skeletal muscle relaxants application and the traditional Low-frequency ventilation.

## Methods

### Study subjects

A total of 427 patients with CAO underwent flexible bronchoscopy or rigid bronchoscopy in the First Affiliated Hospital with Nanjing Medical University from January 2016 to October 2019, of which the clinical data of 375 patients were retrospectively reviewed. Inclusion criteria: (1) Patients with central airway obstruction;(2) need urgent flexible or rigid bronchoscope surgery with various methods for controlling the airway, such as electrocoagulation (including snare electrocoagulation, electrocautery, high-frequency electrosurgical ablation, ect.), electrocautery, cryotherapy, argon plasma coagulation, balloon inflation and stent placement or removal (Table 1); (3) need general anesthesia. Exclusion criteria: (1) patients with intermedius space occupation or lower bronchus stenosis; (2) patients with mediastinal tumors, foreign body; (3) patients with bronchopleural, bronchoesophageal, bronchomediastinal or tracheoesophageal fistula, (4) patients who just underwent biopsy by bronchoscopy; (5) incomplete data or associated anomalies. This study has passed deliberation of the Clinical Ethics Committee of the First Affiliated Hospital with Nanjing Medical University (approval number: 2019-SR-505).

**Table 1:** Patient baseline characteristics

Characteristics	N (%)			*P* value
	Flexible (204)	Rigid (171)	Total (375)	
Age, media (range), year	63 (16 ~ 89)	62 (12 ~ 87)	62 (12 ~ 89)	0.70
Gender (M:F)	115:89	106:65	221:154	0.271
BMI (%)	22.5 ± 3.4	21.8 ± 3.5	22.2 ± 3.4	0.127
ASA				
II ~ III	156 (76.5%)	127 (74.3%)	286 (75.5%)	0.915
IV	44 (21.6%)	39 (22.8%)	84 (22.1%)	
V	4 (2.0%)	5 (2.9%)	9 (2.4%)	
Airway procedures				
Endobronchial stenting	91 (44.6%)	87 (50.9%)	178 (47.5%)	0.226
Balloon dilatation	29 (14.2%)	25 (14.6%)	54 (14.4%)	0.912
Argon plasma coagulation	14 (6.9%)	10 (5.8%)	24 (6.4%)	0.689
Cryoablation	12 (5.9%)	8 (4.7%)	20 (5.3%)	0.605
Endobronchial laser	7 (3.4%)	3 (1.8%)	10 (2.7%)	0.315
Electrocoagulation	89 (43.6%)	78 (45.6%)	167 (44.5%)	0.700
Stent removal	3 (1.5%)	4 (2.3%)	7 (1.9%)	0.536

*BMI* Body Mass Index, *ASA* American Society of Anesthesiologist

### Anesthesia management

General anesthesia was conducted by anesthesiologists. All patients were monitored with Electrocardiograph (ECG), Pulse Oxygen Saturation (SpO_2_), invasive arterial blood pressure (IABP), and given pre-oxygenation with 100% oxygen, 8 ~ 10 L/min for at least 3 min before anesthesia induction. General anesthesia was induced with etomidate or propofol, fentanyl or together with remifentanil, cisatracurium or rocuronium, with or without midazolam, and maintained with propofol and remifentanil. The depth of anesthesia was adjusted according to the intensity of surgical stimulation and hemodynamic indicators.

Patients in flexible group was ventilated by Laryngeal mask airway (LMA) or endotracheal intubation. Patients in rigid group were ventilated by a side port of rigid bronchoscope during the procedure, and LMA insertion or endotracheal intubation was performed immediately after the procedure for sustaining the ventilation. 8-10 L/min pure oxygen was maintained during the whole operation. When the patient’s SpO_2_ dropped below 90%, the operation was stopped and the scope was removed to ventilate the patient. After a few minutes of ventilation, when the patient’s SpO_2_ reached 99–100%, the operation continued. The patients were sent to the recovery room for resuscitation after operation, and muscle relaxant antagonists were given at appropriate time for patients who had no contraindications. Patients with the modified Aldrete score above 9 points were sent back to the general ward, while patients with the modified Aldrete score below 9 points or couldn't be extubated were sent to intensive care unit (ICU).

### Outcome measurements

On the basis of the anesthetic record of each patient, we analyzed the use rate of muscle relaxants, the dosage of muscle relaxants, operation duration, recovery time, artery blood gas, End-tidal carbon dioxide (EtCO_2_), the incidence of perioperative adverse events, postoperative outcomes and the risk factors for patients entering the ICU after surgery. The perioperative adverse events were failure of ventilation, bronchospasm, intraoperative cough, and hypoxemia, hypercapnia, reintubation after extubating. Bronchospasm was defined as wheezing or significantly increased airway pressure during mechanical ventilation, hypoxemia was defined as oxygen saturation < 90%, and hypercapnia is the elevation in the partial pressure of carbon dioxide (PaCO_2_) above 45 mmHg.

### Statistical analysis

SPSS version 23.0 program was used for statistical analysis, and measurement data are expressed as mean ± standard deviation (X ± SD), and counting data are expressed by frequency (n) or rate (%). Analyses are compared between flexible bronchoscopy and rigid bronchoscopy. Chi-square test was used for count data, t-test for measurement data, paired t test for paired groups measurement date, Bivariate Correlation analysis for the correlation between two groups, and Binary Logistic regression analysis for risk factors of postoperative ICU admission. Although the amount of blood gas analysis samples obtained was small (flexible group *n* = 18, rigid group *n* = 17) when reviewing the data, we still performed a correlation analysis of carbon dioxide partial pressure and operation time based on the existing data by Bivariate Correlation analysis. Statistical significance was set at *P* < 0.05, and all tests were two-tailed.

## Results

### Clinical characteristics of included patients

Baseline clinical characteristics of included patients are shown in Table 1. Of the 375 patients with CAO, 204 received flexible bronchoscopy treatments (flexible group) while the other 171 received rigid bronchoscopy treatments (rigid group). As shown in Table 1, there were no significant differences in age, gender, BMI (body mass index), and ASA grades between the two groups (*p* > 0.05). What’s more, one or more airway procedures may be performed in a patient, such as placing a stent and then performing balloon dilation, ect. Endobronchial stenting (44.6% in flexible and 50.9% in rigid) and Electrocoagulation (43.6% in flexible,45.6% in rigid) were the most utilized interventions during the flexible or rigid bronchoscopy (Table 1), and there was no difference in airway procedures between the two groups (*p* > 0.05).

The malignant tumor is the main stenosis pathogen (68.8% in total, 64.4% in flexible and 73.7% in rigid, respectively, *p* > 0.05), and the main cause of CAO for performing flexible or rigid bronchoscopy is primary lung tumor (36.3% in total, 38.7% in flexible and 33.3% in rigid, respectively, *p* > 0.05). Other causal diseases are esophageal cancer, tracheal tumor, scarring, post-placement of stenting, thyroid tumor, lymphoma and tuberculosis (Table 2). The location of CAO in tracheal diagnosed by helical computed tomography (CT) scan or bronchoscopy was 66.7%(68.8% in total, 64.4% in flexible and 73.7% in rigid, respectively, *p* > 0.05), while main bronchus (left or/and right main bronchus) stenosis was 33.3% (Table 2).

**Table 2:** Etiology and location of patients with CAO undergoing flexible bronchoscopy or rigid bronchoscopy

	N (%)			*P* value
	Flexible (204)	Rigid (171)	Total (375)	
Etiology of CAO				
Lung tumor	79 (38.7%)	57 (33.3%)	136 (36.3)	0.418
Esophageal cancer	45 (22.1%)	42 (24.6%)	87 (23.2)	
Tracheal tumor	39 (19.2%)	26 (15.2%)	65 (17.4%)	
Scarring	25 (12.3%)	23 (13.5%)	48 (12.8%)	
Post-placement of stenting	9 (4.4%)	13 (7.9%)	22 (5.6%)	
Thyroid tumor	4 (2.0%)	4 (2.3%)	8 (2.1%)	
Lymphoma	1 (0.5%)	3 (1.8%)	4 (1.1%)	
Tuberculosis	2 (1.0%)	3 (1.8%)	5 (1.3%)	
Benign/Malignant				
Benign	72 (35.3%)	45 (26.3%)	117 (31.2%)	0.062
Malignant	132 (74.7%)	126 (73.7%)	258 (68.8%)	
Location of CAO				
Tracheal	130 (63.7%)	120 (73.2%)	250 (66.7%)	0.187
Left or right main bronchus	74 (36.3%)	51 (29.8%)	125 (33.3%)	

### The use of skeletal muscle relaxants

The safety of skeletal muscle relaxants used in patients with tracheal stenosis is a big challenge for anesthesiologists. In this research, skeletal muscle relaxants were used in 96.5%(362 patients) of the 375 included patients, in which 83.5% (313 patients) were cisatracurium, 12.0% (45 patients) rocuronium and 1.1% (4 patients) atracurium. The use rate of skeletal muscle relaxants was 96.5% (79.5% cistracurium, 15.8% rocuronium, 1.2% atracurium) in rigid bronchoscopy patients, and it was 96.6% (86.8% cistracurium, 8.8% rocuronium, 1.0% atracurium) in flexible bronchoscopy patients (Table 3), and there was no difference between the two groups (*p* > 0.05).

**Table 3:** Utilization rate of skeletal muscular relaxants in patients with CAO undergoing flexible bronchoscopy or rigid bronchoscopy

muscular relaxants	N (%)			*P* value
	Flexible (204)	Rigid (171)	Total (375)	
Cisatracurium	177 (86.8%)	136 (79.5%)	313 (83.5%)	0.224
Rocuronium	18 (8.8%)	27 (15.8%)	45 (12.0%)	
Atracurium	2 (1.0%)	2 (1.2%)	4 (1.1%)	
No recorded	7 (3.4%)	6 (3.5%)	13 (3.5%)	

Although 96.5% of patients with CAO undergoing flexible or rigid bronchoscopy therapy used skeletal muscle relaxants, we found no patients with the failure of ventilation (Table 4). What’s more, no patients suffered bronchospasm or cough, 13 patients (8 in flexible, 5 in rigid) suffered the hypoxemia during the procedure, and two patients (0.05%) were reintubated after awakening due to dyspnea after extubating (sent to ICU after adjusting the position of the bracket). There was no difference between the two groups in perioperative adverse events (Table 4). In addition, the dosage of skeletal muscle relaxants (Cisatracium) used was higher in rigid bronchoscopy compared with flexible bronchoscopy therapy (10.8 ± 3.8 VS 11.6 ± 3.6 mg, respectively, *p* < 0.05). There was no difference between the two groups in procedure duration (41.4 ± 32.5 VS 41.5 ± 29.9 min, respectively, *p* > 0.05) and awakening duration (25.3 ± 21.0 VS 25.8 ± 17.3 min, respectively, *p* > 0.05).

**Table 4:** The comparison of skeletal muscular relaxants and perioperative adverse events between flexible bronchoscopy and rigid bronchoscopy therapy in patients with CAO

Events	N (%)			*P* value
	Flexible (204)	Rigid (171)	Total (375)	
Cisatracium (mg)	10.8 ± 3.8(*n* = 177)	11.9 ± 3.6(*n* = 136)	11.3 ± 3.8	0.008
Procedure duration (min)	41.4 ± 32.5	41.5 ± 29.9	41.1 ± 31.3	0.961
Awakening duration (min)	25.3 ± 21.0(*n* = 179)	25.8 ± 17.3(*n* = 144)	25.6 ± 19.4	0.185
Perioperative adverse events				
Failure of ventilation	0	0	0	1.000
Bronchospasm	0	0	0	1.000
Intraoperative cough	0	0	0	1.000
Hypoxemia	8 (3.9%)	5 (2.9%)	13(3.5%)	0.779
Reintubation after extubation	2 (0.98%)	0	2 (0.5%)	0.503

### Assessment of traditional low-frequency ventilation

**Fig. 1:**
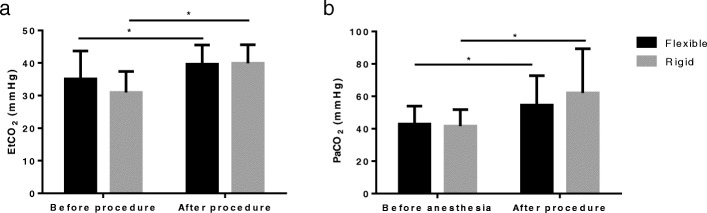
Change in EtCO_2_ and PaCO_2_ before and after therapy in patients with CAO. **a**, Change in EtCO_2_ before and after procedure (*n* = 100 in flexible group and *n* = 57 in rigid group recorded in the anesthesia note). **b**, Change in PaCO_2_ before anesthesia and after procedure (*n* = 18 in flexible group and *n* = 17 in rigid group recorded in the anesthesia note). Paired t test used in these data, *p < 0.05, Flexible: flexible bronchoscopy treatment; Rigid: rigid bronchoscopy treatment

**Fig. 2:**
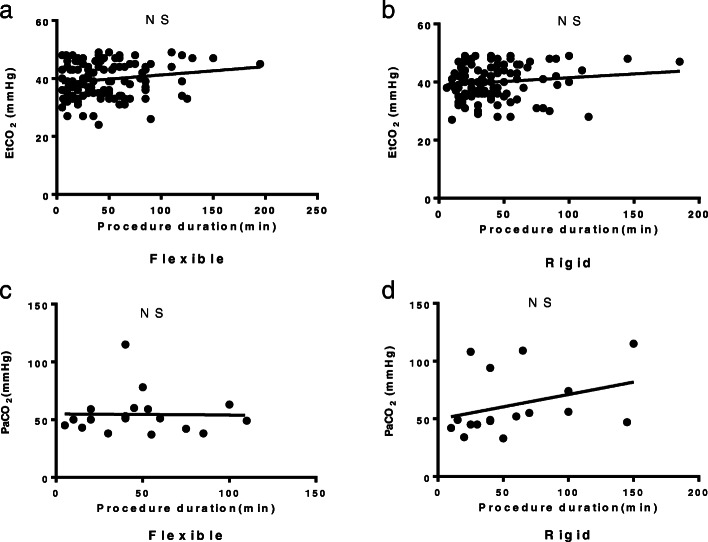
The Correlation between the procedure duration and EtCO_2_ or PaCO_2_ after flexible bronchoscopy or rigid bronchoscopy therapy in patients with CAO. **a**, **b**, The correlation between the procedure duration and EtCO_2_ after flexible bronchoscopy and rigid bronchoscopy therapy (*n* = 133 in flexible group and *n* = 121 in rigid group recorded in the anesthesia note). **c**, **d**, The correlation between the procedure duration and PaCO_2_ after flexible bronchoscopy and rigid bronchoscopy therapy (*n* = 18 in flexible group and *n* = 17 in rigid group recorded in the anesthesia note)

Different from High or Low frequency jet ventilation, the traditional Low-frequency ventilation was used in all patients with CAO. EtCO_2_ and partial pressure of carbon dioxide in artery blood (PaCO_2_) was investigated to evaluate the adequacy of ventilation and gas exchange (Fig. 1). The level of EtCO_2_ in patients after either flexible bronchoscopy or rigid bronchoscopy both increased (35.76 ± 7.71 VS 40.19 ± 6.04 mmHg, 31.72 ± 6.32 VS 37.88 ± 6.15 mmHg, respectively, *p* < 0.05), but the increased extents were not very remarkable (Fig. 1a). The level of EtCO_2_ in the blood gas collected immediately after the operation also increased compared with that before the operation both in two groups (42.25 ± 10.54 VS 55.35 ± 17.54 mmHg, 43.93. ± 13.70 VS 59.50 ± 24.24 mmHg, respectively, *p* < 0.05), that is, most patients suffered hypercapnia during the procedure (Fig. 1b). But the occurrence of hypercapnia has no correlation with the duration of operation both in flexible bronchoscopy and rigid bronchoscopy therapy in patients with CAO (Fig. 2).

Of all patients, only 13(8 in flexible, 5 in rigid) had the lowest SpO_2_ drop below 90%, while 33 (15 in flexible, 18 in rigid) fluctuated between 90 and 95% during the procedure (Table 5). PaO_2_ values were higher in the flexible group than in the rigid group during the therapy, yet both above 200 mmHg (293.2 ± 40.07 vs 204.1 ± 41.03 mmHg), but had no significant difference (*p* > 0.05) .

**Table 5:** Patients with lowest SPO_2_ < 95% and oxygen pressure (PaO_2_) in the arterial blood gas during the therapy (PO_2_: *n* = 18 in flexible group and *n* = 17 in rigid group recorded in the anesthesia note)

	N (%)			*P* value
	Flexible (204)	Rigid (171)	Total (375)	
The Lowest SPO_2_				
< 90%	8 (3.9%)	5 (2.9%)	13 (3.5%)	0.475
90% ~ 95%	15 (7.3%)	18 (10.5%)	33 (8.8%)	
PaO_2_ (mmHg)	293.2 ± 174.66	204.1 ± 158.90	253.9 ± 171.37	0.134

### Post-operative recovery

Following the procedure, 323 patients were sent back to the ward safely after waking up. Fifty-two patients were transferred to ICU due to poor general condition, of which 3 patients died (2 due to hemoptysis and 1 due to acute myocardial infarction) in Day2 after the bronchoscopy therapy (Table 6). The risk factors (including age, ASA, hypertension, diabetes, abnormal ECG, coronary heart disease, lung infection, respiratory failure before procedure, cerebrovascular disease) for patients entering ICU after surgery were conducted in this research. The correlation regression analysis indicated that higher ASA grade and obvious dyspnea or orthopnea were the independent risk factors for postoperative ICU admission (Table 7).

**Table 6:** Post-operative recovery

Outcome	N (%)			*P* value
	Flexible (204)	Rigid (171)	Total (375)	
Ward	177 (86.8%)	146 (85.4%)	323 (86.7%)	0.765
ICU	27 (13.2%)	25 (14.6%)	52 (13.3%)	
Death in 48 h after surgery	1 (0.5%)	2 (1.2%)	3 (0.8%)	0.594

**Table 7:** Binary logistic regression analysis for risk factors for postoperative ICU admission

Risk factors	OR (95% CI)	*P* value
ASA	0.469 (0.283 ~ 0.777)	0.003
Dyspnea or orthopnea	2.878 (1.315 ~ 6.298)	0.008

## Discussion

Central airway stenosis is known worldwide as a life-threatening condition with many causes [9–11]. In this study, we retrospectively reviewed 375 cases with CAO undergoing bronchoscopy with general anesthesia. The causes of CAO were primary tracheal tumors or lung cancer, esophageal cancer, scarring after tracheotomy, post-placement of stenting, mediastinal tumor, pulmonary metastatic tumor, and tracheomalacia etc.. As complications of these diseases, tracheal stenosis can be treated in many ways. Surgery may be the preferred approach, but not all patients are appropriate surgical candidates [4]. Therefore, bronchoscopy treatment remains the best tool for the safest management of airway obstructions, and provides prompt and durable palliation to patients ineligible for surgical treatment [3, 7, 12, 13].

Both rigid and flexible bronchoscopy are now available for the interventional pulmonologists to perform this operation for advanced diagnostic and therapeutic purposes. Flexible bronchoscopy was performed through a laryngeal mask airway or endotracheal tube, which can create auto positive end expiratory pressure and alter airway mechanics with a minimum of sedation. Rigid bronchoscopy relies on the use of a laryngoscope and either a rigid ventilating bronchoscope or Hopkins rod telescope, which can alter the airway by stenting the airway open, and often requires a deeper level of sedation [14]. There are some debates as which one is better than the other, and whether the use of muscle relaxants is safe and indispensable in this procedure [15–20]. In some articles, the authors are in favor of the non-use of muscle relaxants in rigid or flexible bronchoscopy for the safe factor [17, 21], but a recent research showed that controlled ventilation with muscle relaxants during stenting reduced the incidence of desaturation events, maintaining a favorable respiratory status [22]. A rigid bronchoscope can be placed under deep sedation without muscle relaxants, but that required high doses of analgesic and hypnotic agents, which may lead to cardiovascular instability or residual drug effects harming pulmonary function after the operation, and if the depth of anesthesia is not enough, it may causes the trauma of the vocal cords and larynx, even accidental airway perforation, due to the significant response to tracheal manipulation. The use of topical anesthetics is recommended by the ACCP (American College of Chest Physicians) for both basic and advanced bronchoscopy as it reduces the dose of sedative agents needed and effectively decreases cough [23]. Tracheal reflexes are blunted by incorporating a ‘spray-as-you-go’ technique of topical lidocaine spray via the working channel of the bronchoscope [24]. The use of local anesthetics also can be observed in our research, but with the administration of muscle relaxants, which can provide good surgical conditions, the frequency and dose of local anesthetics were not so high.

In our research, we have observed that the use of muscle relaxants can facilitate the placement of rigid bronchoscope, ensure vocal cord adduction, and prevent life-threatening patient moving and coughing during the procedure, thus to provide the best operating conditions. Although SGA (supraglottic airway) insertion itself may not necessitate muscle paralysis, paralyzed vocal cords facilitates bronchoscopy in adduction position. Furthermore, muscle paralysis could attenuate the risk of patient’s coughing and movements during the operation, as well as lower the chest wall resistance and reduce inspiratory pressures needed to achieve satisfactory tidal volumes [25–28]. At the beginning, we also did not dare to use muscle relaxants, but with the improvement of anesthesia equipment, visual technology, and anesthesia skills, we began to experiment with muscle relaxants. Approximately 96.5% of the 375 included patients were given skeletal muscle relaxants as recorded in the anesthesia notes, no patients suffered the failure of ventilation, bronchospasm or cough, only 13 patients (8 in flexible, 5 in rigid) suffered the hypoxemia during the procedure, and two patients (0.05%) were reintubated after awakening due to dyspnea after extubating (sent to ICU after adjusting the position of the bracket). In addition, the dosages of muscle relaxants used in rigid bronchoscopy are significantly higher than those used in the flexible bronchoscopy due to the higher degree of irritation, but that didn’t affect the patient’s awakening. The results may illustrate that the muscle relaxants can be safely used both in flexible and rigid bronchoscopy treatments in patients with CAO, and more dose of muscle relaxants should be given in rigid bronchoscopy treatments.

In this process, the way of mechanical ventilation is also a key factor affecting gas exchange for the patients with CAO undergoing flexible or rigid bronchoscopy treatments. In the past years, high-frequency jet ventilation had become the main ventilation method for bronchoscopy in the treatment of central airway stenosis [29]. A previous study has demonstrated no difference in arterial blood gas analysis values between jet ventilation and conventional ventilation during endobronchial laser surgery, yet jet ventilation may be associated with some complications including hypertension, hypoxemia, hypercapnia, and barotrauma [23]. In this study, the traditional Low-frequency ventilation was used in all patients with CAO. We compared ETCO_2_, PaCO_2_ and PaO_2_ between the flexible and rigid bronchoscopy group to assess whether traditional ventilation can provide adequate ventilation. Many patients with CAO already had hypoxia before surgery, and even 98.4% of the patients experienced symptoms of dyspnea [30]. Therefore, most of them inhaled oxygen when they entered the operating room for emergency bronchoscopy surgery. The SpO_2_ value of most patients was between 93 and 100%, which couldn’t reflect the true hypoxia. In addition, hypoxemia and hypercapnia may commonly occur during bronchoscopic procedures. During the procedure, we noticed SpO_2_ decreased in some patients, despite fraction of inspired oxygen (FIO_2_) being kept at 100%, but no patient suffered severe hypoxemia or hypercapnia. For patients undergoing some transient episodes of SpO_2_ lowering below 90%, high fresh gas flows are often used to obtain adequate ventilation and compensate for the airway leakage. If it didn’t work, we would remove the placed bronchoscope and then ventilate the patient for several minutes until SpO_2_ increased to above 95%, then restart the procedure. PaCO_2_ values were significantly higher than preoperative level in both groups, and most patients suffered hypercapnia during the operation (PaCO_2_ > 45 mmHg), but there was no correlation between the operation time and EtCO_2_ or PaCO_2_ after the procedure both in the flexible group and rigid bronchoscopy group. Different from hypoxemia, hypercarbia is generally well tolerated unless severe enough (above 80 to 100 mmHg) to cause obtundation and respiratory arrest, and moderate hypercarbia may be a favorable condition in a number of pathologic situations [31]. Intraoperative hypercapnia caused by insufficient ventilation can be adjusted by hyperventilation soon after the operation completed. So in this study, there was no obvious life-threatening hypercapnia occurred. The results may show that the Low-frequency traditional ventilation also can meet the adequacy of ventilation and gas exchange in patients with CAO undergoing bronchoscopy therapy. Since some cases have been excluded in our study due to the possible advantages of using HFJV in these cases, including bronchopleural, bronchoesophageal and bronchomediastinal fistulae, we still don’t recommend the routine use of jet ventilation in the procedures described.

In this study, most of the patients with CAO who underwent bronchoscopy therapy were safely transferred to the ward (86.7%), while the others were sent to ICU postoperatively due to their poor general condition. Variables identified as increased complication rate predictors for therapeutic bronchoscopy (including both rigid and flexible) include: emergent procedures, ASA physical status scores [23]. We revealed that the grade of ASA and obvious dyspnea or orthopnea were the independent risk factors for postoperative ICU admission. Therefore, ICU admission may be a safe option when an urgent bronchoscopy is carried out in patients with severe dyspnea, or with high ASA scores. Three deaths (2 due to hemoptysis and 1 due to acute myocardial infarction) occurred during the procedures or within 48 h postoperatively, with a mortality rate of 0.8%. The causes of these three deaths were not directly related to the procedures even though they occurred in the perioperative period. The rest of the patients (99.2%) recovered without incidents in the recovery room in the immediate postoperative period.

There are still some limitations in our study. Firstly, we did not have a blank control group to compare the procedures performed without muscle relaxants. Secondly, a lot of blood gas data were missing from the data during the operation. And thirdly, there was a lack of studies investigating the optimal dosages of muscle relaxants, we will design some prospective researches in the future.

## Conclusions

The muscle relaxants and low-frequency traditional ventilation can be safely used both in flexible and rigid bronchoscopy treatment in patients with central airway obstruction. Given the rise in the interventional therapy, bronchoscopy treatments of CAO under general anesthesia may turn more frequent in the coming future, and this research may provide a safe anesthesia management option for its implementation.

## Data Availability

The data sets used and/or analyzed during the current study available from the corresponding author on reasonable request.

## Abbreviations

CAO: Central airway obstruction
ECG: Electrocardiograph
SPO_2_: Pulse Oxygen saturation
IABP: Invasive arterial blood pressure
LMA: Laryngeal mask airway
ICU: Intensive care unit
EtCO2: End-tidal carbon dioxide
BMI: Body mass index
ASA: American Society of Anesthesiologist
PaO_2_: Partial pressure of oxygen in artery
PaCO_2_: Partial pressure of carbon dioxide in artery
FIO_2_: Fraction of inspired oxygen
SGA: Supraglottic airway
